# Post-Exercise Hypotension: An Alternative Management Strategy for Hypertension and Cardiovascular Disease?

**DOI:** 10.3390/jcm12134456

**Published:** 2023-07-03

**Authors:** Khaled Aly, Pollen K. Yeung

**Affiliations:** College of Pharmacy and Department of Medicine, Faculty of Health and Medicine, Dalhousie University, Halifax, NS B3H 4R2, Canada; kh217984@dal.ca

Cardiovascular disease (CVD), including hypertension, is a leading cause of death worldwide and imposes an enormous burden on our societies. It was estimated that hypertension alone affects 25% of the world’s population, and it is considered one of the most fatal, yet preventable risk factors for cardiovascular disorders and other chronic diseases [1]. The prevalence of hypertension in the adult population is approximately 30–45%. However, in the population over the age of 60 years, it exceeds 60% because blood pressure (BP) increases gradually with age, owing to the hardening of the blood vessels and increased vascular resistance [2]. Partaking in chronic exercise through different activities is widely perceived as an effective strategy that provides various cardiovascular benefits [3]. However, much less is known about the acute or short-term effects of exercise on blood pressure, the mechanism behind blood pressure control and its potential as an alternative prevention strategy for hypertension and CVD.

Post-exercise hypotension (PEH) is a phenomenon describing a prolonged decrease in resting blood pressure in the minutes and hours following acute exercise. Although PEH was first documented by Hill in 1897 during the 90 min following a 400-yard dash [4], it was only after Fitzgerald’s 1981 anecdotal report of the effect of jogging on his own blood pressure that the scientific community began to systematically examine this phenomenon [5]. PEH has been observed after as little as 10 min and as long as 170 min after exercise, although the majority of the studies have only considered endurance exercise lasting between 20 and 60 min. Understanding the cause and effect of PEH may provide a natural (alternative) way of managing hypertension and other CVDs with/without drug therapy [6].

Physical activity and/or exercise such as casual or brisk walking has been shown to effectively delay development of hypertension and reduce blood pressure (BP). Most professional and government organizations now recommend moderate-intensity aerobic exercise for at least 30 min at least 3 days a week or resistance exercise 2–3 days a week. In general, exercise sessions can either be continuous for 30 min or made up of short intervals of at least 10 min for a daily total of 30 min. Regular daily exercise can provide a 5 mmHg decrease in SBP, which could be translated into a 9% reduction in mortality from coronary heart disease, a 14% reduction in mortality due to stroke and a 7% reduction in all-cause mortality [2]. There is evidence to indicate patients with hypertension are more responsive to the post-exercise effect than normotensive individuals [7], suggesting exercise has a stronger and longer-lasting effect on lowering blood pressure in this patient population. A similar observation was also found in animal models when comparing the effect of PEH between spontaneously hypertensive rats (SHR) vs. normotensive Sprague Dawley (SD) rats [8].

Exercise intensity and duration have a significant effect on PEH. It has been shown that while a single session of aerobic plus resistance exercise (RAE) and high-intensity interval exercise (HIIE) can both bring about similar PEH in hypertensive patients with ischemic heart disease (IHD), RAE appears to have significantly less effect on diastolic and atrial functions, which makes it more suitable for reducing blood pressure in hypertensive patients with IHD [9]. Another method of inducing PEH is using sprint interval training (SIT), which has shown that the length of the interval between successive sprints and the age of the subjects can affect their post-exercise response. Specifically, longer resting intervals appear to provide a stronger physiological stimulus, suggesting that resting between successive exercise may in fact produce a greater PEH [10]. However, further studies are needed to investigate the effects of other SIT protocols to determine the optimal resting protocol for maximizing the PEH effect.

Another modified approach is using resistance exercise with blood flow restriction (BFR) to maximize the PEH effect. Although this combination can result in greater PEH than traditional exercise, it also induces higher SBP and/or DBP values compared to traditional exercise, especially in hypertensive individuals. Thus, exercise with BFR should not be used to manage BP control during exercise, e.g., in patients with CVD [11].

In addition to lowering blood pressure, chronic exercise and exercise training can reduce other cardiovascular risk factors, such as diabetes and obesity, etc., and provide a beneficial effect for cardiovascular protection. Exercise has a direct beneficial effect on systemic metabolism and promotes numerous cellular adaptations in the cardiovascular system; its effect extends to other organs and tissues (e.g., liver, skeletal muscle, brain, adipose tissue), which provides a secondary beneficial effect for the cardiovascular system. It has been shown that the sustained post-exercise vasodilation observed in PEH in both healthy individuals and patients with type 2 diabetes (T2D) enhances glucose transport in systemic circulation, which improves glycemic regulation, suggesting a direct linkage between exercise and glucose utilization [12]. Furthermore, chronic aerobic exercise also promotes several positive vascular adaptations, which can attenuate the deleterious effects induced by aging, hypertension, diabetes mellitus or other cardiovascular complications. The process of metabolic adaptation may be attributed to changes in gene and protein expression induced by chronic exercise [13], which are responsible for the prolonged cardiovascular benefits; however, further research is needed to understand the metabolic linkage between chronic exercise and cardiovascular protection.

The mechanism underlying post-exercise hypotension is not completely clear. It is often attributed to a series of autonomic and metabolic events that occur following exercise, including reduced sympathetic outflow and local vasodilator mechanisms [14,15]. Other mechanisms that contribute to PEH include upregulation of nitric oxide synthase in the endothelium as well as the renin–angiotensin system, which occurs in response to exercise [13,16,17]. In one study, exercise increased ATP concentrations in the red blood cells (RBCs) of healthy volunteers immediately after exercise, which may be a surrogate biomarker for increased energy metabolism in response to exercise [18]. We have also shown a similar increase in ATP concentrations in response to exercise in an experimental rat model, and this increase was associated with a cardiovascular protective effect from exercise against acute myocardial infarction [8,16]. The linkage between post-exercise hypotension and ATP was further supported by a recent clinical study that demonstrated a single oral dose of ATP (400 mg) given 30 min before exercise increased the effect of PEH on hypertensive women [19].

During ischemia/hypoxia or in individuals under extremely heavy workloads, such as intense exercise, there is an increased demand of energy, which triggers a rapid breakdown of ATP to ADP and then to AMP and subsequently the release of ADO locally and into the systemic circulation to increase blood and oxygen supply to various organs and tissues. After restoring the hypoxia to normal physiologic conditions, the released ADO is taken up rapidly by endothelial cells and RBCs via nucleoside transporters, and subsequently converted back to ATP by ADO and adenylate kinases. Such a salvage pathway is capable of readily recycling ATP from ADO without depleting intracellular ATP, which is essential for cell survival, even in cells without mitochondria, such as RBCs. It is possible that RBC concentrations of ATP may be an indicator of its concentrations in the myocardium and other tissues. It has been hypothesized that RBCs serve as an oxygen sensor in the cardiovascular system, leading to the release of increased amounts of ATP as the oxygen content falls, and the hemoglobin becomes desaturated. Thus, when RBCs travel through microcirculation, they can release vasodilatory compounds such as ATP and adenosine that enhance blood flow in hypoxic tissues. The released ATP and adenosine help to increase blood supply to the tissue and preserve an optimum balance between oxygen supply and demand, thereby maintaining cardiovascular homeostasis and preserving intracellular ATP in the tissues affected. Such a mechanism would eliminate the requirement for a diverse network of sensing sites throughout the vasculature and should provide a more efficient means of appropriately matching oxygen supply with demand, allowing an immediate switch to alternative energy sources under hypoxia conditions. Thus, it is possible that ATP metabolism in RBCs may be used as a surrogate biomarker for energy content in the myocardium, and perhaps also as a measure of the “inner energy” in the body [8].

Other mechanisms that have been proposed to underlie the effect of chronic exercise and exercise training include attenuation of insulin resistance and endothelial dysfunctions, improving plasma lipid profiles, anti-inflammatory effects, reducing psychological stress, stem cell mobilization and tissue regeneration, release of exerkine, ROS-induced hormesis and autophagy [13]. Some or all these mechanisms may contribute to the cardiovascular protective effect of chronic exercise.

While moderate-intensity aerobic exercise is recommended for at least 30 min 3 times a week and/or resistance exercise 2–3 days a week, which can either be continuous for 30 min or made up of intervals of at least 10 min for a daily total of 30 min [2], and at least 150 min per week of moderate-intensity aerobic activity is recommended by the American Heart Association (AHA) for the adult population (https://health.gov/our-work/nutrition-physical-activity/physical-activity-guidelines/current-guidelines, accessed on 17 June 2023), the clinical health benefits of such exercise programs are still inconclusive. Further research, including confirmation trials, is needed to establish clear clinical guidelines on how specific exercise protocols could prevent/reduce serious cardiovascular outcomes for patients with heart diseases and other chronic illnesses and specific populations, such as obesity, diabetes, heart disease, children, elderly and paraplegic patients [20]. In light of such limitations, specific exercise programs should be developed and applied with close monitoring, especially in patients with cardiovascular diseases.

Altogether, the evidence demonstrates the potential of using exercise to manage hypertension and to protect against cardiovascular events, and suggests that increased ATP metabolism may be an important mechanism for post-exercise hypotension and cardiovascular protection. Concentrations of ATP in RBCs may also serve as a surrogate biomarker for energy metabolism in the body, which is a hypothesis worthy of further research. The beneficial effect of exercise stretches far beyond the cardiovascular system to include other chronic diseases such as musculoskeletal diseases, anxiety and depression, cognitive performance, dementia and Alzheimer’s disease, etc. Compared to pharmacotherapy, physical exercise has no negative side effects, costs very little and targets many health issues at once, which make it an ideal alternative therapy for most non-communicable diseases [21].
